# Unit Costing of Health Extension Worker Activities in Ethiopia: A Model for Managers at the District and Health Facility Level

**DOI:** 10.15171/ijhpm.2017.102

**Published:** 2017-09-02

**Authors:** Maureen E. Canavan, Erika Linnander, Shirin Ahmed, Halima Mohammed, Elizabeth H. Bradley

**Affiliations:** Yale Global Health Leadership Institute, Yale University, New Haven, CT, USA.

**Keywords:** Health Extension Workers (HEWs), Costing Tools, Health System Strengthening

## Abstract

**Background:** Over the last decade, Ethiopia has made impressive national improvements in health outcomes, including reductions in maternal, neonatal, infant, and child mortality attributed in large part to their Health Extension Program (HEP). As this program continues to evolve and improve, understanding the unit cost of health extension worker (HEW) services is fundamental to planning for future growth and ensuring adequate financial support to deliver effective primary care throughout the country.

**Methods:** We sought to examine and report the data needed to generate a HEW fee schedule that would allow for full cost recovery for HEW services. Using HEW activity data and estimates from national studies and local systems we were able to estimate salary costs and the average time spent by an HEW per patient/community encounter for each type of services associated with specific users. Using this information, we created separate fee schedules for activities in urban and rural settings with two estimates of non-salary multipliers to calculate the total cost for HEW services.

**Results:** In the urban areas, the HEW fees for full cost recovery of the provision of services (including salary, supplies, and overhead costs) ranged from 55.1 birr to 209.1 birr per encounter. The rural HEW fees ranged from 19.6 birr to 219.4 birr.

**Conclusion:** Efforts to support health system strengthening in low-income settings have often neglected to generate adequate, actionable data on the costs of primary care services. In this study, we have combined time-motion and available financial data to generate a fee schedule that allows for full cost recovery of the provision of services through billable health education and service encounters provided by Ethiopian HEWs. This may be useful in other country settings where managers seek to make evidence-informed planning and resource allocation decisions to address high burden of disease within the context of weak administrative data systems and severe financial constraints.

## Background

Over the last decade, Ethiopia has made impressive national improvement in health outcomes, including substantial reductions in maternal, neonatal, infant, and child mortality.^1,2^ This success has been attributed in part to the country’s expansive investment in the Health Extension Program (HEP),^3-7^ which has trained and deployed nearly 35 000 health extension workers (HEWs), and established 15 000 health posts, local primary health services facilities, and approximately 2500 health centers since 2003.^8,9^ As the HEP in Ethiopia continues to evolve and improve, understanding the unit cost of HEW services is fundamental to planning for future growth and ensuring adequate financial support to deliver effective primary care throughout the country. Nevertheless, the administrative and financial data needed to support routine costing approaches have been lacking in Ethiopia, as in many resource-limited settings.^10^

A number of costing tools have been created by global development partners.^11,12^ These tools have been used to estimate the total costs of community health worker programs at a national level,^13^ as well as the cost-effectiveness of HEW programs in various country settings^14^; however, these approaches provide high-level perspectives on national costs and are less practical for routine managerial decision-making at the level of individual districts or health centers. For instance, previous studies have not reported estimates for activity-specific costing of HEW services or related potential HEW fee schedules, information that is essential to effectively manage the activities of frontline health workers and to design a sustainable package of primary care services. In Ethiopia, more detailed activity-based costing models could be used by health managers to anticipate costs associated with HEW program expansion or introduction of new services, or to identify and manage the major cost drivers of HEW services relative to local burden of disease. In health systems that allow cost-recovery for HEW services, activity-based costing models can be used by managers and policy-makers to set fee schedules that incorporate both cost and clinical benefit, and by practitioners to optimize revenue. Additionally, cost information may help managers model and anticipate future staffing and budget needs to support expected changes in demand for various types of services, as well as to identify when staff may be misallocated based on expected volume of services.

Accordingly, we sought to examine and report the data needed to generate a HEW fee schedule that would allow for full cost recovery for HEW services. To conduct our analysis, we used data from (1) a time-motion study (TMS) that described how HEWs spend their time in diverse settings,^15^ (2) an exploratory study of Woreda (Ethiopian equivalent of a district)-level costs associated with the HEW program, and (3) a multi-country study that estimated national costs of community health worker programs^13^ (including Ethiopia-specific estimates). Findings from this study can help policy-makers and managers in Ethiopia quantify the costs of HEW service provision, forecast costs associated with projected expansion of HEW scope, and project cost savings associated with increased HEW efficiency. Furthermore, the methods used may be useful in other country settings where managers who lack access to routine administrative data seek to make evidence-based planning and resource allocation decisions to address high burden of disease within severe financial constraints.

## Methods

### Data Sources

#### Health Extension Worker Activity Data

We estimated the time spent in different activities by HEWs in Ethiopia using data from *HEPCAPS II Project. 2015. Health Extension Workers Time Motion Study.*^15-17^ The work activities of two HEWs in 22 Woredas (for a total of 44 HEWs) were observed for a period of 21 consecutive days between April and June 2014. Each day, observers recorded activities continuously beginning with the HEW’s first work-related task of the day upon arrival and ending when the HEW completed the last work-related task of the day before leaving for the day. In addition to the continuous recording of HEW activities, HEWs estimated the time spent on periodic health activities over the past 12 months, including participation in intensive outreach and immunization campaigns, as well as periodic non-health activities such as tax collection and increasing community participation in voting The classification of HEW time was based on another large time motion study in Ethiopia,^18^ and is consistent with time motion studies of community health workers in other settings.^19,20^ For a complete description of the TMS methods and results, please refer to *Ethiopia’s health extension workers use of work time on duty: time and motion study.*^17^

From all activities captured in the TMS, we identified a subset that we termed “billable activities” – that is activities in which the user (either an individual or a community) was identifiable. Examples include, but are not limited to, counseling on family planning, providing a vaccination, or convening a community health education program. Activities that enable billable activities but themselves do not involve service provision to a client or end user, including travel, recordkeeping and recording, or receiving training and supervision, were not included in the list of billable activities. Because the scope of practice differed for urban and rural HEWs, we created separate lists of billable activities for urban and rural areas (Table 1).

**Table 1:** Billable HEW Activities by Urban and Rural Areas

**Urban HEW Activities**	**Rural HEW Activities**
• Hygiene and Environmental Sanitation	• Hygiene and Environmental Sanitation
Family health services • Provide contraceptives • Provide antenatal and postnatal care • Provide care for sick and healthy children (includes newborn) • Vaccinations (Includes TT and Child) • Provide nutrition education/services • Provide health education (education not covered elsewhere)	Family health services • Provide contraceptives • Provide antenatal and postnatal care • Provide care for sick and healthy children (includes newborn) • Vaccinations (Includes TT and Child) • Provide nutrition education/services • Provide health education (education not covered elsewhere)
Disease prevention and control • Provide education/services on HIV/AIDS • Test, educate and provide malaria treatment	Disease prevention and control • Provide education/services on HIV/AIDS • Provide voluntary counseling & testing on HIV • Test, educate and provide malaria treatment • Provide TB related services
• Provide first-aid (includes education and referral)	• Provide first-aid (includes education and referral)
• Non-communicable Diseases	• Non-communicable Diseases
• Extended trainings (Women's Development Army)	• Extended trainings (Women's Development Army)

Abbreviations: HEW, health extension worker; TT, tetanus toxoid; TB, tuberculosis.

For each type of billable activity, we used data from the TMS to calculate the average time spent by an HEW per patient/community encounter (we use the term ‘encounter’ to refer to each discrete time an activity was performed). As is recommended in other time-driven activity-based costing efforts,^21-29^ we estimated the HEW’s allocation of time across daily activities from a larger sample of observations and listed the average experience, recognizing that activity levels vary substantially between days and seasons. We reported the standard deviation on the average times observed in each activity to describe the variation in work patterns. We used the average time conducting an encounter for each billable activity category as the statistic by which we allocated the salary costs.

#### Cost Estimates From Local Administrative Systems

To obtain salary and non-salary cost data at the Woreda level, we identified 7 Woredas from the 22 Woredas included in the TMS, selected to represent the four major regions, urban and rural communities, and higher and lower performing sites in primary care service provision as described previously.^15^ With the goal of identifying and quantifying as many of the costs associated with the HEW program as possible, we deployed an exploratory data collection tool to capture Woreda-level information on annual budgeted and actual costs of primary healthcare service delivery, including HEW and other staff compensation, cost of supplies used by HEWs in provision of services and education, health post infrastructure and maintenance costs, and other costs associated with HEW program operations (eg, travel, housing). We piloted and refined the exploratory tool, and collected data from July 2014 through September 2014. The research team completed the paper-based data collection tool in conjunction with management and finance staff in each Woreda Administration Office were with a standard set of questions asked in local languages (Supplementary file 1). Researchers reviewed finance documents to resolve any discrepancies.

#### Cost Estimates From National Studies

Data on costs for supplies, management (the salary for individuals involved in the HEP that supervised HEWs), and other overhead were not consistently available. To fill this gap, we used an Ethiopia-specific national-level estimate for the proportion of HEW total program costs from a published study on community health worker programs across sub-Saharan Africa.^13^ This estimate noted that salary made up 27% of total costs, management accounted for 9%, other overhead accounted for 13%, and supplies were 55% of total cost. Using this data, we estimated non-salary cost multipliers to calculate the total cost of the Ethiopian HEW program, inclusive of these categories, with the goal of generating an HEW fee schedule that would recover the full costs of the provision of services in the HEP. Consistent with other studies,^13^ labor costs is a relatively small proportion of the total costs of providing HEP primary care services. Subsequently, with only this limited information garnering the cost of all elements that contribute to the program would result in multipliers greater than 1. Using these estimates, we multiplied HEW salary cost by 188% to account for supplies (medical supplies such as vaccines, transportation and office items), an additional 33% to account for the cost of management, and an additional 49% to account for overhead cost. Adding together these three categories necessary for full cost recovery of the provision of services for the HEP was equivalent to multiplying HEW salary costs by 270% (Table 2) and 82% when supplies were not incorporated into total cost.

**Table 2:** Health Education and Service Cost Scale Among Urban HEWs

**HEW-Provided Education and Services in Households and Health Posts**	**Percentage and Duration of Average Encounter** ^a^			**Base Salary Per Encounter**		**Total Cost Per Encounter (82% Non-salary Rate)** ^b^		**Cost Per Encounter (270% Non-salary Rate)** ^c^	
	**Percent Breakdown of Encounters Across 12 HEWs**	**Average Time/Encounter(SD)** ^d^		**Birr**	**US$** ^e^	**Birr**	**US$** ^e^	**Birr**	**US$** ^e^
Hygiene and environmental sanitation education/services	47.1	8.8	(10.6)	16.6	0.75	30.2	1.37	61.4	2.79
Family health services									
Provide contraceptives	1.5	10.7	(3.5)	20.2	0.92	36.8	1.67	74.7	3.40
Provide antenatal and postnatal care	4.7	9.3	(8.2)	17.6	0.80	32.0	1.45	65.1	2.96
Provide care for sick and healthy children (including newborn)	1.1	14.8	(19.7)	27.9	1.27	50.8	2.31	103.2	4.69
Provide vaccinations	6.6	29.2	(29.2)	55.1	2.50	100.3	4.56	203.9	9.27
Provide nutrition education/services	6.4	29.9	(29.3)	56.5	2.57	102.8	4.67	209.1	9.50
Provide other health education	13.9	15.0	(14.9)	28.3	1.29	51.5	2.34	104.7	4.76
Disease prevention and control									
Provide education/services on HIV/AIDS	1.9	25.8	(13.4)	48.7	2.21	88.6	4.03	180.2	8.19
Test, educate and provide malaria treatment	8.3	7.9	(6.6)	14.9	0.68	27.1	1.23	55.1	2.50
First-aid education and referral	1.1	12.8	(9.1)	24.2	1.10	44.0	2.00	89.5	4.07
Screening and education for non-communicable diseases	5.5	13.3	(11.3)	25.1	1.14	45.7	2.08	92.9	4.22
Group training (ie, Women's Development Army)	1.9	52.7	(64.7)	99.5	4.52	181.1	8.23	368.2	16.74

Abbreviations: HEW, health extension worker; SD, standard deviation. ^a^ The percentage of encounters was calculated across the 12 HEWs over 1 month duration. ^b^ 82% non-salary rate includes supervisor salary and other overhead. ^c^ 270% non-salary rate includes supplies, supervisor salary, and other overhead. ^d^ Mean (SD) average time /encounter are reported. Excludes activity times greater than 3 standard deviations from the mean to ensure an accurate and non-skewed representation of the overall sample. ^e^ Asumes an exchange rate of 22 ET Birr to US$1.

### Data Analysis

To develop a fee schedule that would allow for full cost recovery, we sought to allocate HEW program costs (observed HEW salaries plus estimated non-salary costs) across billable activities based on the average frequency and duration of encounters within each activity category.

#### Number and Duration of Encounters

For each billable activity category and separately for urban and rural HEWs, we identified the total number of encounters in our sample, the average length of HEW time spent in an encounter, and standard deviation for the average length of time spent in the encounter. We excluded outliers (less than 1% of encounters) to ensure our estimate was representative of the larger sample and not affected by skewed data. We defined outliers as encounters in which the time spent on the activity was greater than 3 standard deviations away from the mean time for that activity.

#### Cost Per Billable Activity

Base salary per minute. Using the local cost data, we calculated the average HEW salary for both urban and rural regions. If a Woreda reported a range of salaries, we used the midpoint of the range as the input for that Woreda. The resulting average salaries were 1513 birr/month in rural settings and 1419 birr/month in urban settings (US$69 and US$64 respectively; enchange rate of 22 Ethiopan birr per US$ used throughout^30^). We then calculated the average HEW salary per minute of HEW time observed in billable activities. This was calculated as monthly salary divided by average time observed on billable activities per month. On average, HEWs spent 1600 minutes (rural) and 900 minutes (urban) per month on billable activities, resulting in a monthly salary per minute of billable activity estimate of 0.94 birr (US$0.04) for rural HEWs and 1.57 birr (US$0.07) for urban HEWs. In addition to the daily activities observed during the TMS, HEWs are also taken out of their workplace to participate in seasonal, multi-day activities including supporting health sector activities such as measles and polio campaigns, supporting development campaigns and other special initiatives outside of the health sector, and receiving in-service training. The TMS revealed that HEWs spend an average of 9 weeks (or 17.42% of the year) on these seasonal campaigns. To account for the opportunity cost (foregone client encounters) associated with these campaigns, we estimated an 31% increase in the base component of the fee schedule to 1.25 birr (US$0.06) per minute for rural HEWs and 1.88 birr (US$0.09) per minute for urban HEWs. Multiplying this salary per minute by the duration of an average encounter in each billable activity category produced the average salary cost per encounter for that activity.

Adjustment for non-salary costs. To get the full cost per activity, we multiplied the salary cost per activity by 82% to account for situations where only management and overhead costs were needed for full cost recovery of the provision of services of the HEP because in many cases, non-governmental organizations or donors finance supplies. (Management was equivalent to 33% of salary costs and overhead costs were equal to 49% of salary costs.) Additionally, we also estimated the full cost per activity including supply costs. Supplies the most costly proportion of the HEW program accounted for almost double the salary costs and thus we multiplied our salary estimates by an additional 188% to capture this cost making the overall multiplier equivalent to 270% of the salary costs.

## Results

We created separate fee schedules for billable activities in urban and rural settings. For each, we included two estimates of non-salary costs (270% and 82% of salary costs, including and excluding supply costs, respectively). In the urban areas, the HEW fees for full cost recovery (including salary, supplies, and overhead costs) ranged from 55.1 birr for malaria education, testing, and treatment to 209.1 birr for nutrition education and services to 368.2 birr per training session for health development army members (Table 2). The rural HEW fees (again that include salary, supplies, and overhead costs) ranged from 19.6 birr for provision of tuberculosis (TB) services to 219.4 birr for HIV testing and counseling to 460.7 birr for training health development army members (Table 3). We also translated the results of the rural fee schedule into a sample encounter form to promote full cost recovery for rural HEWs (Figure).

**Table 3:** Health Education and Service Cost Scale Among Rural HEWs

**HEW-Provided Education and Services in Households and Health Posts**	**Percentage and Duration of Average Encounter** ^a^			**Base Salary Per Encounter**		**Total Cost Per Encounter (82% Non-salary Rate)** ^b^		**Cost Per Encounter (270% Non-salary Rate)** ^c^	
	**Percent Breakdown of Encounters Across 12 HEWs**	**Average Time/Encounter(SD)** ^d^		**Birr**	**US$** ^e^	**Birr**	**US$** ^e^	**Birr**	**US$** ^e^
Hygiene and environmental sanitation education/services	25.4	12.9	(17.5)	16.2	0.74	29.5	1.34	59.9	2.72
Family health services									
Provide contraceptives	14.2	7.9	(6.7)	9.9	0.45	18.0	0.82	36.6	1.66
Provide antenatal and postnatal care	6.3	20.5	(18.6)	25.7	1.17	46.8	2.13	95.1	4.32
Provide care for sick and healthy children (including newborn)	5.8	15.9	(11.6)	19.9	0.90	36.2	1.65	73.6	3.35
Provide vaccinations	15.4	11.3	(11.5)	14.2	0.65	25.8	1.17	52.5	2.39
Provide nutrition education/services	5.9	18.1	(21.2)	22.7	1.03	41.3	1.88	84.0	3.82
Provide other health education	10.4	14.1	(17.1)	17.7	0.80	32.2	1.46	65.5	2.98
Disease prevention and control									
Provide education/services on HIV/AIDS	0.8	27.8	(41.4)	34.9	1.59	63.5	2.89	129.1	5.87
Provide voluntary counseling and testing on HIV	0.2	47.3	(18.6)	59.3	2.70	107.9	4.90	219.4	9.97
Test, educate and provide malaria treatment	10.1	16.6	(10.3)	20.8	0.95	37.9	1.72	77.0	3.50
First-aid education and referral	1.4	9.2	(7.4)	11.5	0.52	20.9	0.95	42.6	1.94
Provide TB related services	1.9	4.2	(2.4)	5.3	0.24	9.6	0.44	19.6	0.89
Screening and education for non-communicable diseases	1.3	6.1	(5.4)	7.7	0.35	14.0	0.64	28.5	1.30
Group training (ie, Women's Development Army)	1.0	99.3	(77.0)	124.5	5.66	226.6	10.3	460.7	20.94

Abbreviations: HEW, health extension worker; SD, standard deviation; TB, tuberculosis. ^a^ The percentage of encounters was calculated across the 12 HEWs over 1 month duration. ^b^ 82% non-salary rate includes supervisor salary and other overhead. ^c^ 270% non-salary rate includes supplies, supervisor salary, and other overhead. ^d^ Mean (SD) average time /encounter are reported. Excludes activity times greater than 3 standard deviations from the mean to ensure an accurate and non-skewed representation of the overall sample. ^e^ Asumes an exchange rate of 22 ET Birr to US$1.

**Figure:**
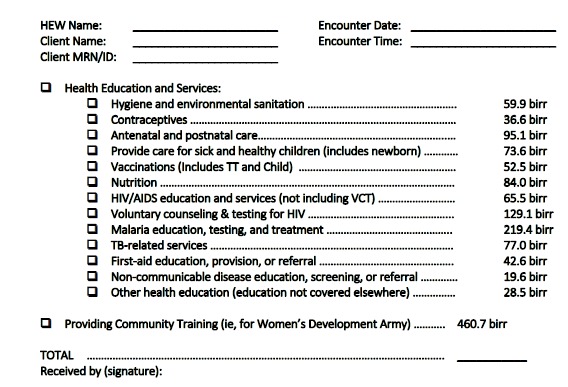
Sample Encounter Sheet for Rural HEWs.
Abbreviation: HEW, health extension worker.

## Discussion

Despite calls for improved managerial capacity and data-driven decision-making at the district and facility level,^31^ efforts to support health system strengthening in low-income settings have often neglected to generate adequate, actionable data on the costs of primary care services. In this study, we have combined time-motion and available financial data (local and national) in Ethiopia to generate a fee schedule for HEW services. The fee schedule allows for full cost recovery through billable health education and service encounters provided by Ethiopian HEWs. The full cost for services ranged from 19.58 birr (US$0.89) for provision of TB services to 219.36 birr (US$9.97) for HIV testing and conseling. The fee schedule also identifies a manageable set of billable activities that are feasible for HEWs to track on a routine basis, generating both productivity data and expected cost recovery data for themselves and their supervisors. To contextualize the fees, the most recent data on Ethiopian per capita income is 12 980 birr (US$590).^32^

The cost accounting undertaken to generate the fee schedules is rarely performed in government-supported health systems in low-income countries. National-level studies show that salaries are only part of HEW program expenditures,^18^ and thus we accounted for two different options of costing that demonstrate full cost recovery: one option where the Woreda must cover its own cost of supplies and one option where supplies are donated. Understanding both the HEW time and non-salary resources needed to deliver HEW services can help in planning sustainable financing schemes for supporting such services, which are often the cornerstone of an effective primary care system.

Although previous research has focused on measuring healthcare quality and access in low- and middle-income countries,^33,34^ understanding healthcare costs is critical for estimating resources needed for sustaining strong health systems. Our study examined the activities of a specific type of healthcare provider (the HEW) and enabled the estimation of HEW fees that would allow for full cost recovery of this provision of service to the health system. Previous efforts to examine costs within Ethiopia have not been based on time motion data and thus may not have produced valid and reliable results. For this project, we sought to document the processes and procedures that were in place for the HEP within this resource-limited setting. We were able to capitalize on existing time-driven observed data for the HEW. Future efforts to apply time-driven activity-based costing (ABC) to improve the process of care delivery by HEWs and limit bottlenecks within the HEP as well as extending costing studies to a broader range of health providers would be useful.

Our results should be interpreted in light of the limitations of the study. Because of the paucity of non-salary cost data, we applied a non-salary cost rate estimate rather than accounting directly for all of the non-salary costs associated with provision of HEW services (supplies, management, and overhead). Less information is available for budgeting and controlling the non-salary costs, which are a substantial portion of the total costs of services. While the system helps identify labor-related costs, it provides less guidance for managers to plan and monitor supply or overhead costs associated HEW services. Applying national-level estimates to the Woreda level may have introduced measurement error; however, the non-salary cost estimates we used were grounded in Ethiopia-specific estimates of HEW program costs, and, for sensitivity analysis, we provided two non-salary cost rate estimates. Second, the gaps in local data limited our ability to assign specific supply costs to their corresponding billable activities (ie, HIV test kits assigned to HIV services), which may have resulted in the underpricing of supply-intensive services and overpricing of services requiring few or no supplies. Nevertheless, when taken as a whole, these fee schedules are designed to allow for full cost recovery at the program level. Last, our approach was limited by a lack of local data on non-salary costs, reducing managerial information about Woreda-level variation in supplies and overhead costs associated with HEW services. Future costing studies that incorporate more detailed Woreda-level supply and overhead costs would provide more nuanced understanding of the sources of differences in Woreda efficiency. The dual investment in more robust accounting systems and increased capacity for using data at the Woreda level would allow for tailored, locally-relevant decision-making.

Our analysis demonstrates the feasibility of using existing data to support managerial decision-making based on cost accounting for primary care services delivered by HEWs. Our approach, which emerged in the context of large gaps in administrative data, is designed for use in resource-limited settings to estimate provider fee schedules that allow for full cost recovery. Findings from this study can help policy-makers and managers in Ethiopia quantify the costs of HEW service provision, forecast costs associated with projected expansion of HEW scope, and project cost savings associated with increased HEW efficiency. Ultimately, the methods used may be useful in other country settings where managers seek to make evidence-informed planning and resource allocation decisions to address high burden of disease within the context of weak administrative data systems and severe financial constraints.

## Ethical issues

The study proposal was reviewed and deemed exempt by the Human Subject Committee of Yale University, New Haven, CT, USA. Additionally, study procedures were reviewed by the Federal Ministry of Health and relevant Regional Health Bureaus in Ethiopia.

## Competing interests

Authors declare that they have no competing interests.

## Authors’ contributions

EL, SA, HM, and EHB were involved in the designing and implementation of the survey instrument. MEC, EL, and EHB analyzed and interpreted the survey data. MEC, EL, and EHB drafted the manuscript. All authors read and approved the final manuscript.

## Supplementary Materials

Supplementary file 1 contains the HEW Costing Data Collection Tool.

## Key messages

**Implications for policy makers**

- Understanding both the health extension worker (HEW) time and non-salary resources needed to deliver HEW services can help in planning sustainable financing schemes for supporting such services, which are often the cornerstone of an effective primary care system.
- Our analysis demonstrates the feasibility of using existing data to support managerial decision-making based on cost accounting for primary care services delivered by HEWs.
- Our approach, which emerged in the context of large gaps in administrative data, is designed for use in resource-limited settings to estimate provider fee schedules that allow for full cost recovery. Findings from this study can help policy-makers quantify the costs of HEW service provision, forecast costs associated with projected expansion of HEW scope, and project cost savings associated with increased HEW efficiency.

**Implications for the public**

The methods used may be useful in other country settings where managers seek to make evidence-informed planning and resource allocation decisions to address high burden of disease within the context of weak administrative data systems and severe financial constraints.
