# Supplementary material for: Unit Costing of Health Extension Worker Activities in Ethiopia: A Model for Managers at the District and Health Facility Level
Source: Int J Health Policy Manag. 2017 Sep 2;7(5):394–401. doi: 10.15171/ijhpm.2017.102 (PMC5953522; doi:10.15171/ijhpm.2017.102)
Supplement: Supplementary file 1 — contains the HEW Costing Data Collection Tool. [file ijhpm-7-394-s001.pdf]

IJHPM
International Journal of Health Policy and Management

HEW Costing Data Collection Tool

*Whenever possible please refer to the **actual costs incurred during the last financial year**, preferably drawn from accounting documents (not staff estimates).*
If some or all of this information is unavailable at the Woreda Health Office, please refer to Woreda Finance/HR/Administrative Offices or Health Center and make a note of the alternative data source for inclusion in the final description of methods.

Survey number: __________
Date: ___________________________
Name of person administering survey: ____
Region: ____
Name of Woreda: ____
Health Center (if Applicable): ____________________
Total Population: __________________

**Section 1: Overall Budget Info**
Total Woreda Health Office Budget received in the last fiscal year:<br><br>Salary:<br>Operating Budget:<br>Training:<br>Drugs:<br>Project:
Total amount requested in the last fiscal year (if different) ____________________________________
How much of the budget is assigned to the health posts/HEW level out of the total amount received?<br>_____________________________________

Region: ____
Name of Woreda: ____
Name of Health Center (If Applicable): ________________________

**Section 2: Salary and Per-Diem Information (from last fiscal year)**
A. Last fiscal year expense for HEW Supervisors Salary: __________<br>Monthly HEW supervisors Monthly Salary: ______________<br># of HEW Supervisors: ________________
B. Last fiscal year expense for HEW Salary (base salary):___________________________<br>Level 3 HEW Monthly Salary: ______________________<br># HEW: ____________________<br>Level 4 HEW Monthly Salary: ______________________<br># HEW: _____________________

C. Reason and value for per-diems paid to the HEWs. Check all activities that apply and note source of data collection. Try to get actual costs rather than estimate.

Activity	Value	Source of information (Woreda Health, Finance Office, HC, other)
**Trainings**		
**Health Related Meetings**		
**Agricultural outreach**		
**Education**		
**Other (Explain)**		

Region: ____

Name of Woreda: ____

Name of Health Center (If Applicable): _________________________

Section 3: Other Non-Salaried Expenses

Did you provide any housing or social services to HEWs in the last fiscal year?

☐ Yes

☐ No

If yes, list service and costs below:

Type	Cost (Monthly)	Annual (Last FY)	Source of Information (Woreda Health, Finance Office, HC, other)
Communication/Mobile Phone			
Housing			
Meals			
Child Support			
Transportation and/or Difficult Area Allowance			
Professional Development Allowance			
Other (Explain)			

Region: ______________________

Name of Woreda: ______________________

Name of Health Center (If Applicable): ______________

Section 4: Infrastructure/Maintenance

Do you have information on costs associated with operations, infrastructure and maintenance of the health post/health center?

☐ Yes
☐ No

If yes, list service and costs below:

Type	Cost Last Fiscal Year	Source of Information (Woreda Health, Finance Office, HC, other)
Building development and maintenance		
Utilities (Rent, Electricity, Water)		
Equipment purchase and maintenance		
Vehicles (ambulance or other)		
Fuel		
**Other (Explain)**		

Region: ______________________

Name of Woreda: ______________________

Name of Health Center (If Applicable): ______________

Section 5: Supplies

Do you provide any supplies (education and/or medical) to HEWs?

☐ Yes
☐ No

If yes, list service and costs below:

Item	Quantity	Frequency (monthly, quarterly, as needed, other)	Unit Cost (actual or best estimate)	Total cost	Verified? (y/n and note source of information)
